# Amorphous chalcogels with local crystallinity

**DOI:** 10.1038/s41467-022-35387-y

**Published:** 2022-12-23

**Authors:** Lijun Yang, Jian Liu

**Affiliations:** Qingdao Institute of Bioenergy and Bioprocess Technology, Chinese Academy of Sciences, Shandong Energy Institute, Qingdao, 266101 China

**Keywords:** Synthesis and processing, Metal-organic frameworks

## Abstract

Chalcogenide aerogels are receiving widespread attention due to their unique properties. Here we comment on a recent work about amorphous Na–Mn–Sn–S chalcogels featuring local structural control, and provide an outlook for the development of chalcogels and the metal-organic sulfide framework.

## Chalcogels as a novel kind of aerogel

In October of 2022, IUPAC released the 2022 Top Ten Emerging Technologies in Chemistry and aerogels have been listed. As one of the latest aerogels to come to the field, the chalcogenide aerogels (chalcogels) have attracted lots of attention and expanded the applications from the conventional area to some specific fields due to their emerging unique properties^1^. Specifically, the three-dimensional random porous network features a highly polarizable and Lewis-basic porous surface. The amorphous nature of the chalcogels was derived from the spontaneous metathesis reaction between the chalcogenide cluster anions and linker counterions. However, the lack of precise control of the porous chalcogels structure was a bottleneck and the local order in the chalcogels was remaining neglected. It is worth mentioning that nanostructuring the chalcogels was rendered possible by in-situ gel formation on the underlying substrates and the structural hierarchy leads to chalcogels with superaerophocity and superhydrophilicity properties^2^. The microscopic local order, however, was not taken into serious consideration in the previous chalcogels studies.

## Putting order into disorder

Kim and colleagues reported a novel route to realize the multiscale structural control over the chalcogels (figure 3 in ref. 3.). Specifically, by using the manganese linker and thiostannate motif, the crystalline layer structure assembly could be induced in the resultant Na–Mn–Sn–S chalcogels via slow gelation process^3^. Varying the Mn amounts, the structural transformation control could be accomplished from crystalline to amorphous nature. Interestingly, the Na-Mn–Sn–S chalcogels with [Sn_2_S_6_]^4−^:Mn^2+^ ratio (1:0.5) were identical to the KMS-1 (K_2*x*_Mn_*x*_Sn_3−*x*_S_6_) in terms of chemical composition and structural pattern, while the latter was usually obtained by high temperature and pressure hydrothermal synthesis^4^. By further stoichiometrically increasing Mn amounts in the precursor solutions, the more amorphous and porous framework with higher meso- and microporosities could be developed in the resultant chalcogels. The chalcogels could demonstrate the good ion-exchange capacity like the KMS-1 counterpart, although the crystalline structure could be collapsed during the ion-exchange process. Previously, Kanatzidis and colleagues have pioneered the construction of biomimetic chalcogels by introducing the ordered Fe_4_S_4_ and Mo_2_Fe_6_S_8_ clusters into the amorphous framework and the resultant amorphous gel gave fair good performance in terms of N_2_ reduction and H_2_ evolution^5^.

The order-by-disorder arrangement could be extended to the more generalized system in the synthesis of organic linker containing chalcogenide framework. Recently, Anderson and colleagues reported a completely disordered Ni tetrathiafulvalence tetrathiolate coordination polymer with local ordered microdomain, which demonstrates notable conductivity as high as 1200 S cm^−1^ ^6^. Such order-by-disorder finding could abandon the periodic limitations for the further development of conducting organic materials and also bring new applications for the materials. Therefore, amorphous metal sulfide framework with local structural order is worth to be investigated and could bring more unexpected results.

## Outlook

The work by Kim and colleagues suggests that there is direct connection between crystalline framework and amorphous chalcogels^3^. A porous chalcogenide framework materials, featuring the self-assembly of two-dimensional building block in a highly active three-dimensional interconnected network, will arouse great attention in the near future. There is plenty of room at the bottom for further diversifying the structures and compositions of chalcogels. Inspired by the versatile metal-organic framework chemistry, the connection between the metal chalcogenides and the thiolate linkers for forming the sulfur-containing coordination polymer framework was gaining momentum recently since the versatile chalcogenide chemistry was relatively unexplored in the framework materials field^7–9^. The strong and stable bonds between the metal and the sulfur-based bridging ligands render the highly crystalline samples challenging^10^, while the synthesis of amorphous counterpart with local crystallinity could bring new horizon for the community. Given the diversity and unique properties of metal-sulfur clusters and versatile linkers, the importance of amorphous and crystalline metal chalcogenide framework materials is poised to grow in the near future.
